# Ongoing Oscillatory Electrophysiological Alterations in Frail Older Adults: A MEG Study

**DOI:** 10.3389/fnagi.2021.609043

**Published:** 2021-02-18

**Authors:** Isabel Suárez-Méndez, Stefan Walter, David López-Sanz, Natalia Pasquín, Raquel Bernabé, Ernesto Castillo Gallo, Myriam Valdés, Francisco del Pozo, Fernando Maestú, Leocadio Rodríguez-Mañas

**Affiliations:** ^1^Laboratory of Cognitive and Computational Neuroscience (Complutense University of Madrid - Universidad Politécnica de Madrid), Center for Biomedical Technology (CTB), Universidad Politécnica de Madrid (UPM), Madrid, Spain; ^2^Department of Structure of Matter, Thermal Physics and Electronics, Complutense University of Madrid (UCM), Madrid, Spain; ^3^Department of Experimental Psychology, Complutense University of Madrid (UCM), Madrid, Spain; ^4^Foundation for Biomedical Research, University Hospital of Getafe, Getafe, Spain; ^5^Centro de Investigación Biomédica en Red Fragilidad y Envejecimiento Saludable (CIBERFES), Spain; ^6^Department of Medicine and Public Health, Rey Juan Carlos University, Madrid, Spain; ^7^Department of Psychobiology and Methodology in Behavioral Sciences, Universidad Complutense de Madrid (UCM), Madrid, Spain; ^8^Radiology Service, University Hospital of Getafe, Getafe, Spain; ^9^Geriatric Service, University Hospital of Getafe, Getafe, Spain; ^10^Centro de Investigación Biomédica en Red en Bioingeniería, Biomateriales y Nanomedicina (CIBER-BBN), Spain

**Keywords:** frailty, neuroimaging, magnetoencephalography, electrophysiology, aging

## Abstract

**Objective:** The role of the central nervous system in the pathophysiology of frailty is controversial. We used magnetoencephalography (MEG) to search for abnormalities in the ongoing oscillatory neural activity of frail individuals without global cognitive impairment.

**Methods:** Fifty four older (≥70 years) and cognitively healthy (Mini-Mental State Examination ≥24) participants were classified as robust (0 criterion, *n* = 34) or frail (≥ 3 criteria, *n* = 20) following Fried's phenotype. Memory, language, attention, and executive function were assessed through well-validated neuropsychological tests. Every participant underwent a resting-state MEG and a T1-weighted magnetic resonance imaging scan. We performed MEG power spectral analyses to compare the electrophysiological profiles of frail and robust individuals. We used an ensemble learner to investigate the ability of MEG spectral power to discriminate frail from robust participants.

**Results:** We identified increased relative power in the frail group in the mu (*p* < 0.05) and sensorimotor (*p* < 0.05) frequencies across right sensorimotor, posterior parietal, and frontal regions. The ensemble learner discriminated frail from robust participants [area under the curve = 0.73 (95% CI = 0.49–0.98)]. Frail individuals performed significantly worse in the Trail Making Test, Digit Span Test (forward), Rey-Osterrieth Complex Figure, and Semantic Fluency Test.

**Interpretation:** Frail individuals without global cognitive impairment showed ongoing oscillatory alterations within brain regions associated with aspects of motor control, jointly to failures in executive function. Our results suggest that some physical manifestations of frailty might partly arise from failures in central structures relevant to sensorimotor and executive processing.

## Introduction

Frailty is one of the major expressions of cumulative age-related decline that may precede disability in older adults, affecting 10–17% of the aged population (Collard et al., 2012). So far, frailty has been characterized through two main conceptual frameworks: as a predictive factor of adverse outcome in old people, and as the phenotypic expression of the combined effect of natural aging and chronic conditions. The complex pathophysiology of frailty has led to several controversies concerning the domains that integrate the syndrome, and the systems and organs involved in its development. In this regard, the participation of the central nervous system (CNS), and particularly brain structure and function, is one of the queries fueling debate. Several authors propose that the CNS is implicated in the pathophysiology of frailty, specifically causing the cognitive decline that some patients experience alongside the physical manifestations of the syndrome (Ávila-Funes et al., 2009). Accordingly, a particular subtype of frailty, *cognitive frailty*, was introduced in order to subsume those cases in which cognitive and physical disruptions coexist (Kelaiditi et al., 2013). However, even if frailty seems to be highly predictive of cognitive impairment and dementia (Rosado-Artalejo et al., 2017b), most frail patients do not exhibit significant cognitive impairment when assessed through general screening tools like the Mini-Mental State Examination (MMSE) (García-García et al., 2011). In view of this, other authors argue that CNS dysfunction is another manifestation of frailty's multisystem vulnerability, and as such, it should not be detached into specific subtypes. In practice, even if current clinical instruments for diagnosing frailty focus on testing physical items, they might also reveal expressions of brain dysfunctionality beyond cognition (Rodríguez-Mañas and Sinclair, 2014).

Despite the debate, the number of studies investigating the role of the CNS in frailty is still scarce (López-Sanz et al., 2018). Till now, only a handful of neuroimaging reports have identified brain structural (Newman et al., 2001; Buchman et al., 2014; Jung et al., 2014; Avila-Funes et al., 2017; Del Brutto et al., 2017; Kant et al., 2018; Maltais et al., 2020) and functional (Lammers et al., 2020; Suárez-Méndez et al., 2020) abnormalities associated with frailty as a unified entity. In general terms, these studies provided concurring evidence of white matter and ischemic damage in frail individuals. On the other hand, several studies connected CNS structural and functional disruptions to alterations in individual items commonly used to assess frailty, such as gait speed (Rosano et al., 2010; Yuan et al., 2015; Rosario et al., 2016) and handgrip strength (Seidler et al., 2015; Hirsiger et al., 2016). The absence of a more expanded literature is disconcerting considering the successful application of neuroimaging techniques to other fields of research [e.g., dementia (Jagust et al., 2006; Nakamura et al., 2018)] that could be mirrored in the study of frailty. In this work, we used magnetoencephalography (MEG) to investigate the existence of abnormalities in the ongoing oscillatory neural activity of frail individuals without global cognitive impairment. The aim of this work was to delve into the hypothesis that the CNS is involved in the pathophysiology of frailty in the absence of cognitive symptoms and that accordingly, changes in brain function should be observed in frail individuals without significant cognitive impairment that could suggest mild cognitive impairment or dementia. Additionally, we explored whether MEG power spectral properties could be used to train a classifier into discriminating frail from robust participants.

## Materials and Methods

### Participants

The sample of this study was recruited from an outpatient basis of the Geriatric Service of the Getafe University Hospital (Madrid, Spain). One hundred and seventy candidates qualified in the clinical records as frail or robust were originally contacted by phone and invited to participate. For each frail individual, two robust peers of the same age (± 1 year) and gender were included at the invitation stage. Candidates were classified as frail (if meeting ≥ 3 criteria) or robust (if meeting 0 criteria) according to Fried's phenotype criteria (Fried et al., 2001; García-García et al., 2011), namely involuntary weight loss (≥4.5 kg in the last year), self-reported exhaustion, sedentary behavior, poor handgrip strength, and reduced gait speed (Table 1). In order to maximize the findings of our study, and taking into account the sample size, those candidates classified as pre-frail (i.e., those meeting 1–2 criteria) were not considered for the study and excluded. Sixty older adults (robust and frail) were finally enrolled. All the participants were right-handed and native Spanish speakers.

**Table 1:** Diagnostic criteria of frailty.

**Fried's frailty phenotype criteria**	**Assessment**
Exhaustion	Assessed using the responses (YES/NO) to two statements of the Center for Epidemiologic Studies Depression Scale (CES-D) (Orme et al., 1986)
Weight loss	Unintentional weight loss of 4.5 kg in the last year
Reduced physical activity	Assessed by the short version of the Minnesota Leisure Time Activity Questionnaire (Taylor et al., 1978)
Slowness	Assessed by the time to walk 4.3 meters and stratified by gender and height
Weakness	Assessed by handgrip strength and stratified by gender and body mass index

*Frailty was identified by the presence of three or more criteria. Robustness was identified by the absence of all criteria*.

During the initial screening session, an expert neuropsychologist (NP) evaluated the general cognitive status of all the pre-selected participants using the MMSE. For those participants achieving an MMSE score ≥ 24, the following domains were further assessed: (i) memory, (ii) language, (iii) attention, and (iv) executive function. Memory was evaluated with the Logical Memory Score (immediate and delayed recall) from the Wechsler Memory Scale-III (WMS-III, Spanish version), and the Rey-Osterrieth Complex Figure (ROCF). Language function was evaluated with the Boston Naming Test (BNT) and the Phonemic and Semantic Fluency Tests from the Controlled Oral Word Association Test (COWAT). Finally, attention and executive function were evaluated with the Trail Making Test parts A and B (TMTA and TMTB) and the Digit Span Test (forward and backward) of the WMS-III. The neuropsychological assessment was conducted on a single session, and completion times ranged from 43 to 57 min. Additionally, a functional evaluation including the Barthel Index for Activities of Daily Living (ADL) and the Short Physical Performance Battery (SPPB) was performed. The presence of comorbidities was also recorded for all participants.

#### Inclusion and Exclusion Criteria

Participants were required to be at least 70 years of age and to score ≥ 24 in the MMSE (indicative of normal cognition). Furthermore, participants were excluded from our study in case of (i) history of psychiatric disorders, neurological disorders, or drug consumption that could affect MEG activity (e.g., cholinesterase inhibitors), (ii) having a medical condition with increased risk of associated cognitive symptoms, (iii) severe head injury with loss of consciousness within the last 5 years, (iv) usual intake of alcohol (> 3 alcoholic beverages per day), (v) use of neuroleptics, major narcotics, sedative-hypnotics, or anticonvulsants, (vi) hearing or vision impairments that would preclude testing, and (vii) Axis I psychiatric diagnoses including depression. Participants with a history of stroke were excluded only if showing focal neurological symptoms in the neurological exam or a focal impairment in the magnetic resonance imaging (MRI) scan. Finally, individuals with pacemakers or metallic implants that could interfere with MEG or MRI were also excluded.

**Figure 1:**
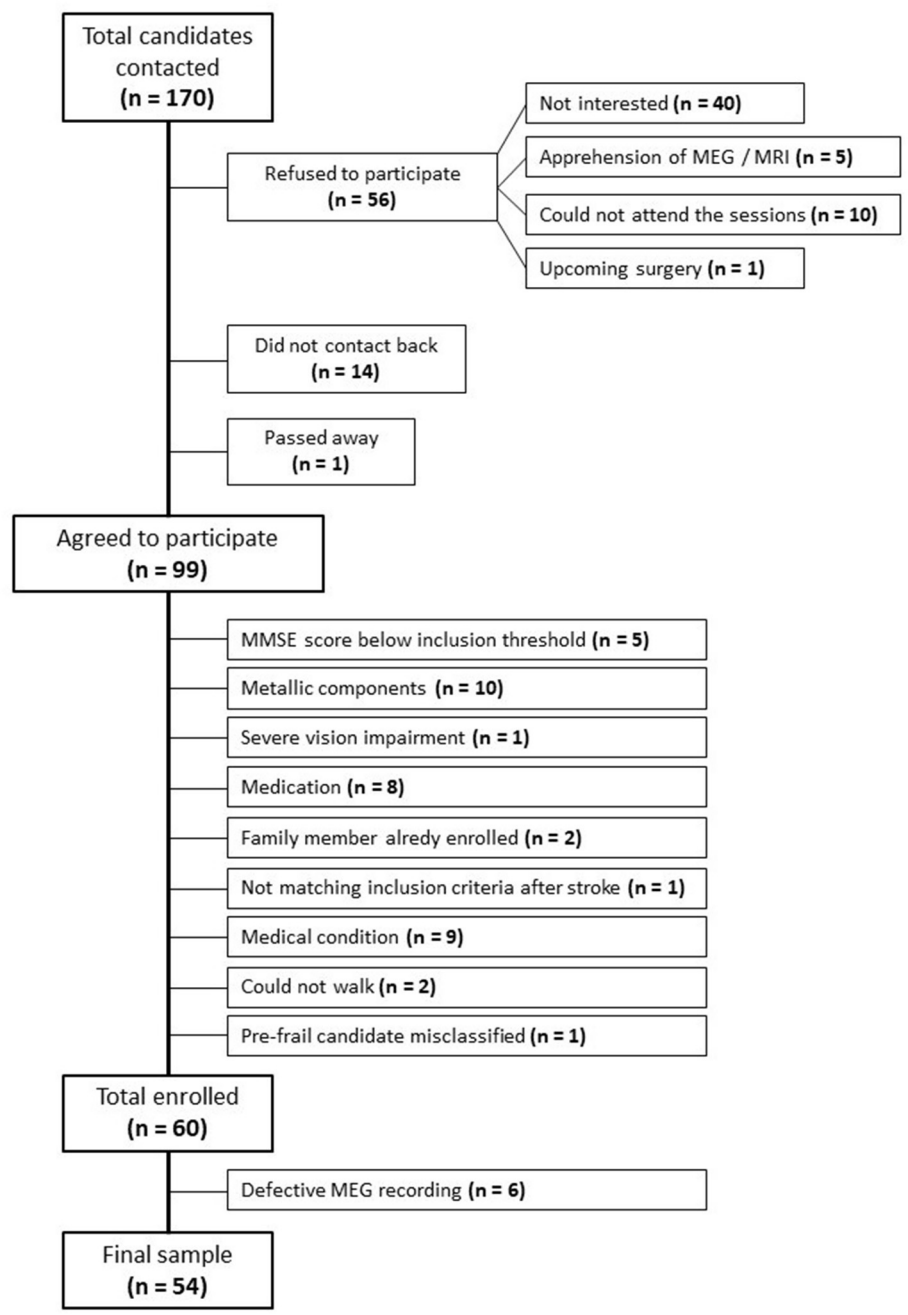
Flow diagram of study participants.

Out of the initial 170 candidates contacted, 56 refused to participate in the study: 40 were not interested after being informed of the proceedings, 5 refused due to fear of MEG or MRI, 4 lived away and could not attend the sessions, 6 were caretakers of dependent adults or children and could not attend the sessions, and 1 candidate had an upcoming surgery. Fourteen candidates did not contact back. One candidate had passed away. Among those favorable to enrolling, 5 candidates scored below the MMSE inclusion threshold, 10 candidates presented pacemakers (1), metallic implants (6), prosthesis (1), or stents (2). One candidate was excluded due to severe vision impairment, 1 candidate was excluded because he was pre-frail and had been misclassified, 2 candidates could not walk, 8 candidates were excluded due to their prescribed medication, and 9 due to diverse medical conditions. Two candidates were excluded because they had family members in the study, and 1 candidate was excluded due to not matching the inclusion criteria after a stroke. This information is summarized in the flow diagram depicted in Figure 1.

### MEG Signal

#### Recordings

MEG data were acquired at the Laboratory of Cognitive and Computational Neuroscience (UCM-UPM) in the Center for Biomedical Technology (CTB) (Madrid, Spain) with a 306 channel (102 magnetometers, 204 planar gradiometers) Vectorview MEG system (Elekta AB, Stockholm, Sweden) situated in a magnetically shielded room (VacuumSchmelze GmbH, Hanau, Germany). The experimental procedure was carefully explained to all the participants upon their arrival at the Center. During the initial setup, we used a 3D Fastrak digitizer (Polhemus, Colchester, Vermont) to identify three fiducial points (nasion and bilateral preauricular points) and to outline the head shape of the participants by digitizing ~400 scalp points. We placed two Head Position Indicator (HPI) coils in the mastoids, and two in the forehead of the participants. The exact position of the HPI coils was acquired with the Fastrak digitizer to allow for head position estimation during the recording. Finally, we placed two electrooculogram (EOG) electrodes (above and below the left eye) to capture eye blinks and ocular movements, and two electrocardiogram (EKG) electrodes (forming a diagonal across the chest) to capture the electrocardiographic signal. After the initial setup, the participants were conducted to the MEG room where they were asked to sit comfortably and still throughout the recording. We recorded 5 min of electrophysiological activity while the participants rested awake with their eyes closed. MEG data were acquired with a sampling rate of 1,000 Hz and an online anti-alias band-pass filter between 0.1 and 330 Hz. The recordings were processed offline using a tempo-spatial filtering algorithm (tSSS) (Taulu and Hari, 2009) (Maxfilter software v 2.2, correlation limit = 0.9, correlation window = 10 s) to eliminate magnetic noise coming from outside of the head. The same algorithm was used to compensate for the movements of the head during the recording.

#### Pre-processing

We identified ocular, muscular, and jump artifacts within our data using the automatic artifact-detection algorithm of the Fieldtrip toolbox (Oostenveld et al., 2011) for Matlab (R2017b, Mathworks, Inc.). Afterward, a MEG expert visually inspected the data to verify the automatic detection. An algorithm based on independent components was used to remove the contributions of EOG and EKG signals from our data. Finally, the remaining data were segmented in epochs of 4 s of artifact-free activity: 77.5 [12.1] trials [mean (SD)] in the frail group, 81.0 [13.1] trials in the robust group, with no significant group-level effect regarding the number of trials (*p* = 0.33). Because of the high redundancy found in MEG data after spatial filtering, only magnetometers' data were used in our analyses (Garcés et al., 2017).

### MRI Acquisition

Following the MEG recordings, a three-dimensional T1-weighted anatomical MRI was acquired for each participant at the Magnetosur clinic in Getafe (Madrid, Spain) with a 0.23 T open configuration (C-shaped) Philips Panorama MRI scanner (repetition time/echo time = 470/16 ms, flip angle 90°, slice thickness = 5 mm, 256 × 256 matrix, and field of view = 270 mm). The T1-weighted images were used for MEG source reconstruction. Additionally, the T1-weighted images were inspected by an experienced radiologist (ECG) to discard the presence of small vessel disease within our sample.

### Source Reconstruction

The clean epochs were band-pass filtered between [2–30] Hz to remove both low-frequency noise and the power line artifact. Before filtering, epochs were padded with 2 s (2,000 samples) of real signal on both ends to avoid edge effects within the data. The source model consisted of 2,459 sources placed in a homogeneous grid of 1 cm in a Montreal Neurological Institute (MNI) template, converted to subject-space by an affine transformation. Each source was assigned to one of the 64 cortical areas defined in the Harvard-Oxford atlas. The lead field was calculated with a single-shell head model (Nolte, 2003) generated from the individual T1-weighted image using the Fieldtrip toolbox (Oostenveld et al., 2011). We employed a Linearly Constrained Minimum Variance (LCMV) beamformer (Van Veen et al., 1997) to obtain the source-space time-series using the lead field and building the beamformer filters with the epoch-averaged covariance matrix.

### Spectral Analysis

We used the beamformer filters to reconstruct the epoched source-space time-series for each cortical source. Afterward, we calculated the MEG power spectra for every clean epoch using the Fast Fourier Transform (FFT) with a Hanning window for frequencies [2–30] Hz and frequency steps of 0.25 Hz. The resulting power spectra were averaged across epochs and normalized by the overall power in [2–30] Hz, thus obtaining the values of the relative power for each frequency step, source, and participant. We searched for group-level effects in the classical frequency bands: theta [4–8] Hz, alpha (mu) [8–13] Hz, and beta [12–30] Hz, and hypothesized further differences in human sensorimotor frequencies (SMR) [12–15] Hz.

### Statistical Analysis

The frail and robust groups were matched for age and gender. Independent samples *t*-tests were used to perform group comparisons in the power-spectral analyses. The elevated number of statistical comparisons in the power spectral analyses made it necessary to apply two procedures for multiple comparisons correction. (1) We performed a Montecarlo permutation method based on the clustering of spatially contiguous significant sources (100,000 permutations, alpha = 0.05). The exact details of the cluster-based permutation test (CBPT) performed herein can be found in the original formulation by Maris and Oostenveld (2007). This method was performed using the Fieldtrip toolbox (Oostenveld et al., 2011) for Matlab (R2017b, Mathworks, Inc.). (2) To correct for the different frequency bands [theta, alpha (mu), beta, and SMR] we used the False Discovery Rate (FDR; Benjamini and Hochberg, 1995) (*Q* = 0.1). This method was performed using Matlab (R2017b, Mathworks, Inc.). For the statistical comparison of functional and neuropsychological scores, we used Fisher's exact test and Mann–Whitney *U*-test for continuous variables.

### Discrimination Analysis

We performed the classification analysis after randomly splitting the study sample into a 2/3 discovery sample and a 1/3 validation sample to gain insight into the external validity. To test the ability of MEG spectral power to correctly classify frail and robust participants, we used an ensemble learner as implemented in R 3.5.1 (R Core Team, 2017) and the SuperLearner package (Polley et al., 2018). This ensemble combined three machine learning algorithms: Least Absolute Shrinkage and Selection Operator (LASSO) [as implemented in the *glmnet* package (Friedman et al., 2010)], a random forest classifier (as implemented in *randomForest*), and a support vector machine (SVM) classifier [as implemented in *e1071* (Meyer et al., 2015)]. We used the default criteria for all classifiers. Because of the large number of variables (258, i.e., age, sex, and 4 frequency bands × 64 cortical areas) compared to the low number of participants, we included a screening step using LASSO for the random forest and the SVM classifiers, and the univariate Pearson correlation *p* < 0.1 for the LASSO classifier for variables to be included in the learner. In the ensemble learner, we maximized the Area Under the Curve (AUC) using leave-one-out cross-validation for model selection. We used the predicted probability of being frail from SuperLearner for Receiver Operating Characteristic (ROC) curve analysis and report the associated AUC as a measure for discrimination quality.

### Ethical Issues

This study was approved by the Ethical Committee of the University Hospital of Getafe (Madrid, Spain). All participants provided signed informed consent prior to study enrollment.

## Results

The sample of our study originally consisted of 60 participants, 6 of which were discarded for the analyses due to problems in the recorded signals. We did not observe significant group differences regarding age (*p* = 0.104), gender (*p* = 0.381), nor comorbidities. Frail participants obtained significantly poorer scores in the ADL [frail: 92.50 (80.00, 100.00) vs. robust: 100.00 (100.00, 100.00); *p* < 0.001] and the SPPB [frail: 7.00 (5.00, 8.00) vs. robust: 10.00 (9.00, 11.75); *p* < 0.001). As expected from the exclusion criteria, mean MMSE was over 24, with no significant differences between groups (*p* = 0.543). We found that frail participants attained significantly poorer results than robust counterparts in some neuropsychological tests examining executive function. Specifically, frail participants performed worse in TMTB (errors) [frail: 4.00 (3.00, 6.00) vs. robust: 2.00 (1.00, 4.75); *p* = 0.032) and the Digit Span Test (forward) [frail: 6.00 (4.75, 7.00) vs. robust: 7.00 (6.00, 8.00); *p* = 0.036]. Additionally, we observed significant group differences in the ROCF (immediate recall) [frail: 8.50 (4.62, 12.50) vs. robust: 12.50 (8.62, 15.50); *p* = 0.039], ROCF (delayed recall) [frail: 8.00 (2.75, 11.62) vs. robust: 11.00 (8.50, 14.00); *p* = 0.021], and the Semantic Fluency Test [frail: 10.50 (9.00, 14.25) vs. robust: 14.00 (11.00, 17.75); *p* = 0.045], indicating lower performance of the frail group. A summary of the demographic, clinical, functional, and neuropsychological information of the final sample can be found in Table 2.

**Table 2:** Demographic, clinical, functional, and neuropsychological assessment data for each study group (robust and frail).

	**Robust**	**Frail**	***p*-value**
*n*	34	20	
Age median [IQR]	78.50 [74.00, 82.00]	81.00 [77.75, 82.75]	0.104
Gender *n* [% female]	21.00 [61.80]	15.00 [75.00]	0.381
**Comorbidities** ***n*** **[%]**			
Hypertension	24 [70.58]	11 [55.00]	0.376
Dyslipidemia	11 [32.35]	6 [30.00]	0.873
Diabetes Mellitus	7 [20.58]	5 [25.00]	0.743
COPD/asthma	7 [20.58]	3 [15.00]	0.727
Osteoporosis, vertebral fracture, low Vitamin D	11 [32.35]	9 [45.00]	0.393
Other (AF, IHD, cancer, incontinence)	16 [47.05]	15 [75.00]	0.053
**Functional status median [IQR]**			
ADL	100.00 [100.00, 100.00]	92.50 [80.00, 100.00]	**<0.001**
SPPB	10.00 [9.00, 11.75]	7.00 [5.00, 8.00]	**<0.001**
**Neuropsychological assessment median [IQR]**			
**Memory**			
Logic memory (I.A)	6.00 [5.00, 7.00]	5.00 [4.00, 6.25]	0.213
Logic memory (I.B)	7.00 [4.00, 10.75]	5.00 [4.00, 11.00]	0.739
Logic memory (II.A)	2.00 [0.00, 5.00]	0.50 [0.00, 3.25]	0.347
Logic memory (II.B)	5.00 [3.00, 7.75]	4.50 [0.75, 6.25]	0.427
ROCF (copy)	28.50 [25.00, 32.00]	29.00 [25.50, 31.00]	0.936
ROCF (immediate recall)	12.50 [8.62, 15.50]	8.50 [4.62, 12.50]	**0.039**
ROCF (delayed recall)	11.00 [8.50, 14.00]	8.00 [2.75, 11.62]	**0.021**
**Language**			
BNT	15.00 [15.00, 15.00]	15.00 [14.00, 15.00]	0.298
Phonemic fluency	21.00 [14.00, 27.50]	20.00 [15.50, 25.00]	0.641
Semantic fluency	14.00 [11.00, 17.75]	10.50 [9.00, 14.25]	**0.045**
**Attention and executive function**			
TMTA (time)	93.00 [69.25, 131.50]	116.50 [69.75, 195.50]	0.119
TMTA (errors)	0.00 [0.00, 1.00]	1.00 [0.00, 1.25]	0.175
TMTB (time)	307.50 [198.50, 379.00]	395.50 [282.00, 604.00]	0.130
TMTB (errors)	2.00 [1.00, 4.75]	4.00 [3.00, 6.00]	**0.032**
Digit span (forward)	7.00 [6.00, 8.00]	6.00 [4.75, 7.00]	**0.036**
Digit span (backward)	6.00 [5.00, 7.00]	6.00 [5.00, 6.00]	0.424
**Cognitive status**			
MMSE	27.00 [26.00, 28.00]	26.00 [25.00, 28.00]	0.543

*COPD, Chronic Obstructive Pulmonary Disease; AF, Atrial Fibrillation; IHD, Ischemic Heart Disease; ADL, Barthel Index for Activities of the Daily Living; SPPB, Short Physical Performance Battery; ROCF, Rey-Osterrieth Complex Figure; BNT, Boston Naming Test; TMTA, Trail Making Test Part A; TMTB, Trail Making Test Part B; MMSE, Mini-Mental State Examination; IQR, Interquartile range. Gender was compared with Fisher's exact test, while the Mann–Whitney U-test was used for continuous variables. For the functional and the neuropsychological assessment higher values are indicative of improved performance for all tests except for the TMTA and TMTB (time and errors). Bold p-values denote statistical significance at the p < 0.05 level*.

Among those who were classified as frail, 9 participants (45%) met 3 criteria, 10 (50%) met 4 criteria, and only 1 participant fulfilled the 5 criteria of Fried's phenotype. Concerning the prevalence of each criterion, fatigue (95%) and reduced handgrip strength (85%) were the most common, while involuntary weight loss (25%) was the least prevalent (Table 3).

**Table 3:** Frail participants (number and percentage) meeting each of the criteria of Fried's frailty phenotype.

**Fried's frailty phenotype criteria**	**Frail participants meeting each criterion *n* [%]**
Exhaustion	19 [95.00]
Weight loss	5 [25.00]
Reduced physical activity	8 [40.00]
Slowness	13 [65.00]
Weakness	17 [85.00]

On another note, evidence of small vessel disease or major axonal decay was not observed in the MRI exams for any of the participants.

### Differences in Relative Power

Significant group differences in relative power were found in the classical alpha (mu) and SMR frequency bands. We found no significant group differences in theta nor beta frequencies after multiple comparisons correction.

#### Alpha (mu) Frequency Band

**Figure 2:**
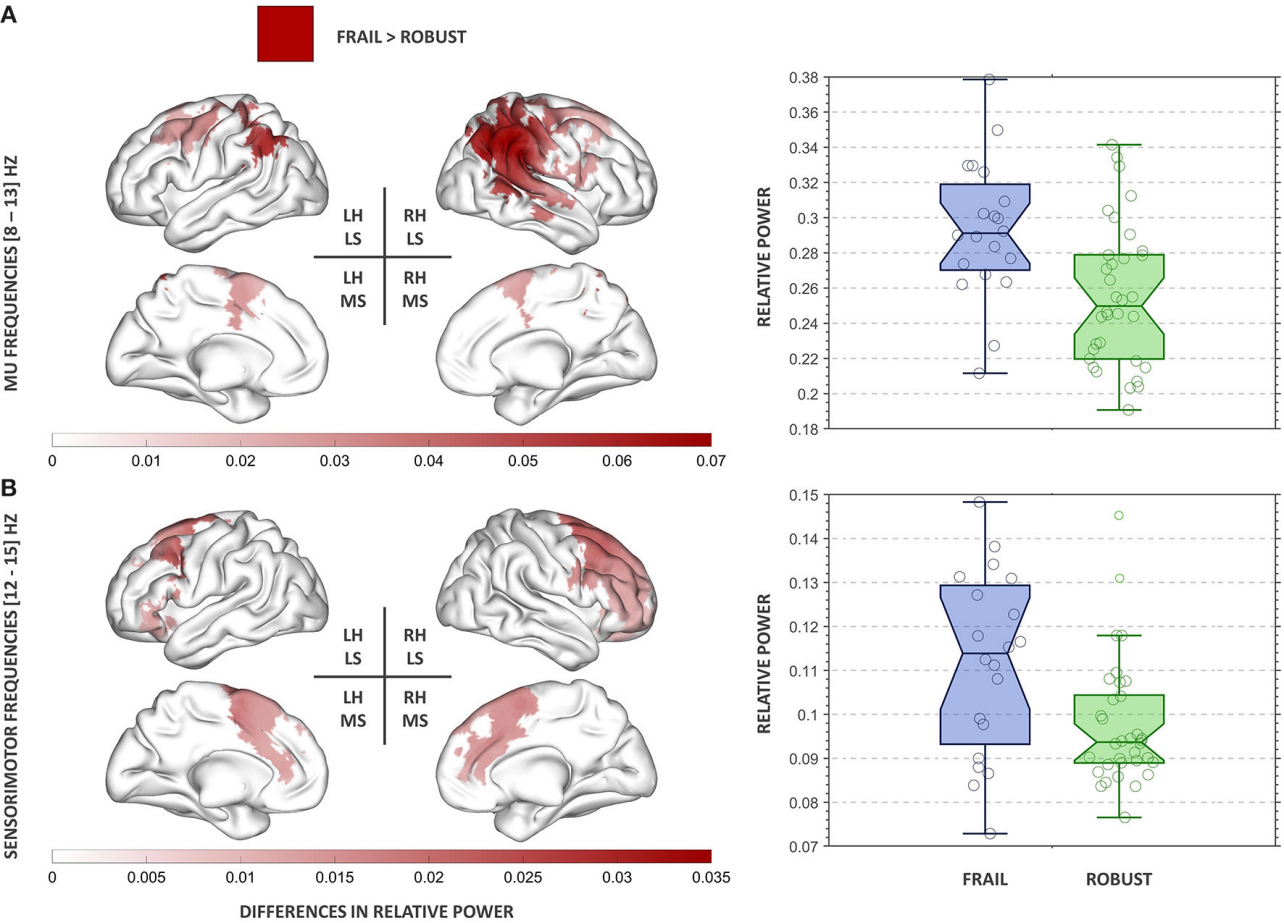
Results of the power spectral analysis in the mu **(A)** and SMR **(B)** frequency bands. On the left panel, clusters showing significant group differences in relative power are displayed for each frequency band. The red colormap indicates the strength of the increase in relative power observed in the frail group. On the right panel, the group relative power distributions for each cluster are presented. The boxplots inform of each participant's relative power value, the median, the 95% confidence interval of the median, and the boundaries of the quartiles. RH, right hemisphere; LH, left hemisphere; LS, lateral surface; MS, medial surface.

We found significant group differences in alpha (mu) relative power after multiple comparisons correction (*p* = 0.02). Frail participants exhibited increased alpha (mu) relative power in comparison to their robust peers. The strongest differences were found in the right hemisphere (RH) affecting posterior parietal areas [e.g., supramarginal gyrus (SMG), angular gyrus (AG), and superior parietal lobe (SPL)], the primary somatosensory cortex, and the superior occipital cortex. Significant bilateral differences involved areas of the primary motor cortex (Figure 2A). These regions comprise those classically associated with traditional generators of mu activity (Pineda, 2005), thus henceforth, we will refer to our results within this range as mu oscillations.

#### SMR Frequency Band

We found significant group differences in SMR relative power after multiple comparisons correction (*p* = 0.03). Frail participants exhibited increased SMR relative power in comparison to their robust peers. The strongest differences were found in frontal areas of the RH, including the premotor cortex, the supplementary motor area, and the paracingulate gyrus (Figure 2B).

### Classification Analysis

To assure a valid classification and reduce the likelihood of overfitting using SuperLearner, we split the whole sample of 54 participants into a 2/3 discovery sample (*n* = 35, 11 frail, 24 robust) and 1/3 validation sample (*n* = 19, 9 frail, 10 robust). When we applied the algorithm obtained from this ensemble to the testing sample, we obtained an AUC = 0.73 (95% CI_Delong_ = 0.49–0.98) which was borderline significant (*p* = 0.083). Using 5-fold cross-validation in the training sample the best machine learning classifier was the ensemble learner with an average AUC of 0.655.

## Discussion

The comparison of the ongoing oscillatory neural activity of frail and robust individuals revealed significant differences in the mu and SMR frequency bands. Frail individuals exhibited increased relative power across sensorimotor, posterior parietal, and frontal areas predominantly localized in the RH. Additionally, frail individuals demonstrated poorer performance in the executive domain. Moreover, an ensemble learner showed promising results in the ability to correctly discriminate between frail and robust participants using MEG power spectral properties.

The analysis of group differences in relative power revealed highly RH lateralized increases in the oscillatory neural activity of frail participants. Frail participants exhibited increased mu relative power across right posterior parietal areas (e.g., SMG, AG, SPL) expanding to the sensorimotor cortex (precentral and postcentral gyri) and the superior occipital lobe. Group differences were most accentuated in the rSMG. This area of the somatosensory association cortex seems to be involved in several functions, from visual word recognition (Stoeckel et al., 2009) to verbal working memory (Deschamps et al., 2014). Importantly, rSMG seems to be involved in the processing of sensorimotor information. Different fMRI studies have revealed consistent SMG activations in response to proprioceptive-related stimuli (Naito, 2004a; Goble et al., 2012; Ben-Shabat et al., 2015), suggesting that the SMG might be a key region for the central processing of proprioception (Ben-Shabat et al., 2015). This proposing is further supported by lesion studies in post-stroke patients connecting damage to the SMG with persisting difficulties in the percept of limb position and movement (Findlater et al., 2018). These results are particularly meaningful considering the importance of accurate limb representations for the correct processing of motor commands and the carrying out of daily routines. Indeed, some of the physical issues observed in frailty (e.g., reduced gait speed, recurrent falls, decline in daily activities) could draw from deficiencies within internal representations that might challenge motor control in frail individuals.

The aforementioned suggestion is consistent with concurring evidence that connects several of the areas that showed abnormal oscillatory activity in the frail group, with a neural network subserving the representation of internal estimates of the body and its parts. Differences in mu relative power spread from the rSMG onto the rAG, rSPL, and sensorimotor cortex. The importance of the SPL in sensorimotor integration is largely documented, particularly concerning the maintenance of updated body estimates (Wolpert et al., 1998; Buneo and Andersen, 2006; Parkinson et al., 2010). The sensorimotor cortex has been associated with the processing of proprioception and kinesthesia (Naito, 2004a), and specifically with the somatic perception of limb movement (Naito et al., 2002; Naito, 2004b). Additionally, the rAG is thought to engage in further aspects of body representation (Spitoni et al., 2013), motor control (Farrer et al., 2008), and near-space perception (Bjoertomt, 2002). Following this, alterations in the oscillatory activity within this network might underlie deficiencies in proprioceptive-related processes in frail individuals. Different intervention programs have demonstrated positive results after incorporating (or focusing on) proprioceptive training in order to confront some of the physical manifestations of frailty (Sohn and Kim, 2015; Tarazona-Santabalbina et al., 2016; Pérez-Ros et al., 2020). The present study provides electrophysiological evidence to support these approaches. In general terms, the central processing of proprioception has been presented as an RH-dominated mechanism (Naito, 2004a; Goble et al., 2006; Goble and Brown, 2007), which might also explain why oscillatory abnormalities appear to be consistently RH lateralized in frail individuals.

The differences in relative power between frail and robust participants were restrained to the mu and SMR frequency bands. Mu oscillations arise across sensorimotor regions in the absence of movement or movement-related imagery. During movement, mu oscillations are suppressed by the desynchronizing activity of sensorimotor neurons leading to a power reduction in the mu range (Pineda, 2005). Mu suppression is thought to reflect sensorimotor processing within fronto-parietal areas and has been widely studied in the context of the human mirror neuron system (Hobson and Bishop, 2016). The extent up to which movement-related activity suppresses mu synchronization seems to be proportional to the peak power values attained at rest (Van Leeuwen et al., 1978). In consequence, the aberrant mu hypersynchrony observed in frail individuals at rest might negatively impact mu suppression during movement planning and execution. An analogous proposing could apply to SMR oscillations that also occur under circumstances of sustained immobility (Sterman, 1996). SMR activity is maximally detected across brain regions neighboring the motor strip, albeit our results showed a cluster of increased SMR relative power in the frail group spreading from right posterior frontal areas, including the premotor cortex and the supplementary motor area, to the anterior prefrontal cortex. Ample evidence connects these regions with mechanisms of gait (Jor'dan et al., 2017), particularly under demanding conditions [also reliant on neurovascular coupling (Jor'dan et al., 2020)] [e.g., high speed (Suzuki et al., 2004), walking while talking (Holtzer et al., 2011), obstacle negotiation (Mirelman et al., 2017), and precision stepping (Koenraadt et al., 2014)]. Additional functions of the prefrontal cortex include balance control (Mihara et al., 2008) and executive function (Seniów, 2012; Barbas and García-Cabezas, 2016). Prefrontal recruitment seems to increase with age, both during normal and complex walking conditions (Mirelman et al., 2017). In the latter case, higher prefrontal activation has been associated with increased gait variability in older adults, possibly suggesting a compensatory effect in the face of complex motor demands (Mirelman et al., 2017). These deficiencies might be intensified in frail individuals showing abnormal oscillatory neural activity across these regions and generalized walking difficulties. According to recent research, non-invasive interventions in the anterior prefrontal cortex might be used as a means of improving gait mechanisms in such functionally-limited populations (Manor et al., 2018).

Even though frail participants showed a slightly decreased mean performance in several cognitive tests, we did not observe significant differences in their cognitive status. None of the frail participants in our study could classify as suffering cognitive impairment attending to their neuropsychological assessment, even if they demonstrated a significantly reduced performance in some specific neuropsychological tests when compared to their robust peers. Frail participants attained lower scores in measures of executive function such as the TMTB and the Digit Span Test (forward), and also in the recall trials of the ROCF and the Semantic Fluency Test, the latter also connected with processes of executive control (Shao et al., 2014). The reduced executive performance observed in the frail group is admittedly in agreement with previous reports associating poor executive function with an increased risk of functional decline, disability, and frailty (Bell-McGinty et al., 2002; Rosado-Artalejo et al., 2017a). Specifically, performance in the TMT has been acknowledged as an independent predictor of mobility impairment, decline in lower limb function, and mortality in cohorts of older adults (Vazzana et al., 2010). Frail participants also scored worse in the recall (immediate and delayed) trials of the ROCF but not in the copying task, suggesting that lower performance in the ROCF was in this case not mediated by executive function but by additional visuomotor memory deficiencies in the frail group.

This work serves as an explorative approach to increase our understanding of the role of the CNS in the development of frailty from an electrophysiological perspective. As such, it suffers from including only a limited number of participants sourced from a single study setting. This shortcoming makes our cohort a non-representative sample of the general population of older age, however, it also guarantees that frailty was assessed homogeneously in the study. The reduced sample size limited the possibility of adjusting the analyses for every desirable variable (e.g., socio-economic conditions, educational level), nevertheless, the potential bias introduced by these sources should be reduced as participants were recruited from a single hospital that serves a specific neighborhood characterized by very similar socio-economic and educational profiles. Frail and robust participants were comparable in terms of comorbidities and, as expected, functional performance was worse in frail participants. In addition, it is important to mention that our results concerned frail individuals that were classified within Fried's phenotype framework. Consequently, our findings cannot be automatically extrapolated to frail samples classified within a different conceptual framework (i.e., accumulation of deficits or biopsychosocial models) whose definition of frailty incorporates items related to neurological or cognitive deficits explicitly excluded in our study.

As a whole, our findings reveal the presence of abnormalities in the ongoing oscillatory neural activity of frail individuals within well-defined areas of the brain mediating proprioceptive and motor processes. Alterations within these central mechanisms, jointly to further peripheral factors commonly found in frail individuals (e.g., sarcopenia), might contribute to several of the physical manifestations generally recognized as the physical components of frailty (i.e., loss of gait speed, handgrip strength, physical activity, and balance). The sample of our study only included participants that were free of global cognitive impairment. Bearing this, our results suggest that frail individuals that could have been framed under the definition of *physical frailty* show evidence of brain dysfunction, also in the absence of substantial anatomical injury. Accordingly, the use of *frailty* as a recognizable clinical entity with different pathophysiological substrates, including alterations in brain function, is in our opinion, most appropriate than its subdivision into specific subtypes. In addition, all the participants were free of ischemic cerebral lesions, another potential, although controversial, pathophysiological factor involving the CNS into generating frailty without cognitive symptoms (Kant et al., 2019). All things considered, our results seem to support the view that the CNS contributes to the unfolding of the so-called physical phenotype of frailty in patients without global cognitive impairment by a mechanism different from ischemic, microvascular lesions.

## Data Availability Statement

The datasets used and/or analyzed during the current study are available from the corresponding author on reasonable request.

## Ethics Statement

The studies involving human participants were reviewed and approved by University Hospital of Getafe, Getafe, Spain. The patients/participants provided their written informed consent to participate in this study. Written informed consent was obtained from the individual(s) for the publication of any potentially identifiable images or data included in this article.

## Author Contributions

IS-M carried out the MEG studies, the analysis of the data, and participated in the writing of the first draft and the final version of the manuscript. SW was involved in the general design of the study, coordinated its implementation, and made substantial contributions to the analyses and writing of the manuscript. DL-S participated in the analyses of the data and the writing of the manuscript. NP, RB, and MV were responsible for the recruitment of the participants, their functional and clinical phenotyping, the design of the psychological assessment, and the clinical groundwork and logistics. EC was involved in interpreting the MRI images. FM, FP, and LR-M were involved in the conception, design, supervision, and analysis of the data. They also participated in the writing of the first draft and the final version of the manuscript. All authors contributed to the article and approved the submitted version.

## Conflict of Interest

The authors declare that the research was conducted in the absence of any commercial or financial relationships that could be construed as a potential conflict of interest.
